# Long non‐coding RNA MALAT1/microRNA 125a axis presents excellent value in discriminating sepsis patients and exhibits positive association with general disease severity, organ injury, inflammation level, and mortality in sepsis patients

**DOI:** 10.1002/jcla.23222

**Published:** 2020-04-20

**Authors:** Wei Liu, Feng Geng, Li Yu

**Affiliations:** ^1^ Department of ICU The Central Hospital of Wuhan Tongji Medical College Huazhong University of Science and Technology Wuhan China

**Keywords:** disease severity, inflammation, long non‐coding RNA MALAT1/micro RNA 125a axis, mortality, sepsis

## Abstract

**Objective:**

The present study aimed to investigate the potential value of long non‐coding RNA metastasis‐associated lung adenocarcinoma transcript 1 (lnc‐MALAT1)/microRNA (miR)‐125a axis in disease management and prognosis surveillance of sepsis.

**Methods:**

Totally, 196 sepsis patients and 196 healthy controls were enrolled. Blood samples were collected within 24 hours after admission in sepsis patients and were collected at enrollment in healthy controls. The relative expression of lnc‐MALAT1 and miR‐125a in all participants was detected by reverse transcription quantitative polymerase chain reaction, and the inflammatory cytokines in plasma of sepsis patients were measured by enzyme‐linked immunosorbent assay.

**Results:**

Lnc‐MALAT1/miR‐125a axis was increased in sepsis patients compared with healthy controls (*P* < .001) and was of excellent value in distinguishing septic patients from healthy controls with the area under the curve (AUC) of 0.931 (95% CI: 0.908‐0.954). In sepsis patients, lnc‐MALAT1 was negatively associated with miR‐125a, and lnc‐MALAT1/miR‐125a axis was positively correlated with acute pathologic and chronic health evaluation II (APACHE II) score, Sequential Organ Failure Assessment (SOFA) score, serum creatinine, C‐reactive protein, tumor necrosis factor‐α, interleukin (IL)‐1β, IL‐6, and IL‐8, while negatively associated with albumin. Furthermore, lnc‐MALAT1/miR‐125a axis was of value in predicting increased 28‐day mortality risk to some extent (AUC: 0.678, 95% CI: 0.603‐0.754).

**Conclusion:**

Lnc‐MALAT1/miR‐125a axis presents excellent value in differentiating sepsis patients from healthy controls and also exhibits positive association with general disease severity, organ injury, inflammation level, and mortality in sepsis patients.

## 1 INTRODUCTION

Sepsis is defined as a life‐threatening organ dysfunction caused by a dysregulated host innate immune response to infecting pathogen.[1] Sepsis is considered to be a leading cause of mortality and a major public health concern, accounting for significant healthcare utilization.[2, 3] Currently, common sepsis management includes use of antibiotics, infection control, fluid resuscitation with crystalloids, appropriate ventilator use, vasopressors, and lactate clearance; however, sepsis remains the primary contributor of death from infection due to the delay of diagnosis.[4, 5] Therefore, the discovery of novel biomarkers is important for facilitating earlier identification and initiating disease management timely in clinical intervention of sepsis.

Long non‐coding RNA metastasis‐associated lung adenocarcinoma transcript 1 (lnc‐MALAT1) is a highly conserved nuclear non‐coding RNA, which is reported to regulate the secretion of inflammatory cytokine in immune and inflammatory system and is correlated with lipopolysaccharide (LPS)‐stimulated cell apoptosis, which leads to the inflammation injury and several organ dysfunctions.[6, 7, 8, 9] Furthermore, based on the miRanda database and the previous studies reported, miR‐125a serves as one of the lnc‐MALAT1 target genes, and lnc‐MALAT1/miR‐125a axis is indicated to regulate the development and progression of several tumors.[10, 11] In addition, the involvement of miR‐125a in sepsis pathology has been reported by previous studies.[12, 13] Mechanically, miR‐125a regulates the development of neutrophil and exerts the anti‐inflammation effect via suppressing pro‐inflammatory factors production via mediating NF‐κB signaling, participating to the innate host defense in sepsis.[13, 14, 15, 16] Clinically, miR‐125a is negatively associated with biochemical index level, disease severity scale scores, and pro‐inflammation cytokine level in sepsis patients.[15] According to these evidences, we hypothesized that lnc‐MALAT1/miR‐125a axis might have increased value in discriminating sepsis patients from healthy controls and have correlation with disease severity, inflammation level, and mortality of sepsis, and part of data about the role of lnc‐MALAT1 in sepsis has been published in the previous study.[17] However, the implication of lnc‐MALAT1/miR‐125a axis in sepsis management has not been studied before. Therefore, we performed the present study to detect the potency of lnc‐MALAT1/miR‐125a axis in distinguishing sepsis patients from healthy controls and further investigate the correlation of lnc‐MALAT1/miR‐125a axis with general disease severity, organ injury, inflammation level, and survival profile in sepsis patients.

## 2 MATERIALS AND METHODS

### 2.1 Participants

One hundred and ninety‐six sepsis patients who admitted in The Central Hospital of Wuhan were continuously recruited as study objects. The recruiting period ranged from January 2017 to June 2019. All patients were diagnosed as sepsis according to the diagnosis criteria proposed in the Third International Consensus Definitions for Sepsis and Septic Shock (Sepsis‐3).[18] Patients above 18 years old, without hematological malignancies or solid tumors and without human immunodeficiency virus (HIV) infection, were eligible for inclusion. And patients died within 24 hours after admission or received immunosuppressive therapy within 1 month before enrollment were excluded. Pregnant or lactating women were also excluded from this study. A control cohort including 196 healthy subjects was enrolled as healthy controls, and the healthy controls were required to have no history of sepsis or other severe infections, no history of malignancies, and no obvious abnormal indexes confirmed by healthy examination.

### 2.2 Ethics

The Ethics Committee of The Central Hospital of Wuhan approved this study, and all procedures were carried out referring to the Declaration of Helsinki. Written informed consent was obtained from each participant or corresponding guardian.

### 2.3 Date collection

Baseline characteristics of sepsis patients including demographic characteristics and common biochemical indexes were recorded after the informed consents were provided. The disease severity and organ dysfunction severity of sepsis patients were evaluated using acute pathologic and chronic health evaluation II (APACHE II) score and Sequential Organ Failure Assessment (SOFA) score, respectively. The demographic characteristics and common biochemical indexes of healthy controls were also collected after they were enrolled.

### 2.4 Sample collection

Blood samples of sepsis patients were collected by peripheral venipuncture within 24 hours after admission, and blood samples of healthy controls were obtained by peripheral venipuncture on the enrollment. After the blood samples were centrifuged at 3000 *g* for 10 minutes (4°C), the plasma samples were isolated and kept frozen at −80°C until assayed.

### 2.5 Inflammatory cytokines measurement

The inflammatory cytokines in plasma of sepsis patients were measured by enzyme‐linked immunosorbent assay (ELISA), which included TNF‐α, interleukin‐1β (IL‐1β), IL‐6, and IL‐8. The procedures were performed referring the instruction of ELISA kits (Thermo Fisher Scientific). In brief, firstly, the plasma samples were added to pre‐coated 96‐well plate to bind the immobilized antibody on the wells. Then, a second antibody was added to the wells to form a sandwich. Finally, a tetramethylbenzidine substrate solution was added to produce measurable signal, and after stop solution was added, the intensity was measured at 450 nm wavelengths on a microplate reader (BioTek).

### 2.6 Lnc‐MALAT1 and miR‐125a detection

For sepsis patients and healthy controls, the relative expression of lnc‐MALAT1 and miR‐125a in plasma was detected by reverse transcription quantitative polymerase chain reaction (RT‐qPCR). Total RNA was extracted from plasma using QIAamp RNA Blood Mini Kit (Qiagen) and then reversely transcribed using iScript™ cDNA Synthesis Kit (with random primer) (Bio‐Rad). Following that, qPCR was performed using QuantiNova SYBR Green PCR Kit (Qiagen) to quantify expression of lnc‐MALAT1 and miR‐125a. The qPCRs were performed triplicated with internal coefficient variation of 1.3% in sepsis patients and 0.8% in healthy donors. The expression level of lnc‐MALAT1 and miR‐125a was calculated using 2^−ΔΔ^ *^C^* ^t^ method with GAPDH and U6 as an internal reference, respectively. The detailed description of normalization was as follows: (a) qPCR was performed in triplicate, and the average of miR‐125a *C*_t_ and U6 *C*_t_ in every sample were determined, respectively. (b) Calculations of Δ*C*_t_ (*C*_t__avg.miR‐125a_ − *C*_t__avg.U6_) were presented in every sample, which was shown as Δ*C*_t__(sample)_. (c) The median of Δ*C*_t_ in healthy controls was referred as the calibrator, which was shown as Δ*C*_t__(calibrator)_. (d) ΔΔ*C*_t_ = Δ*C*_t__(sample)_ − Δ*C*_t__(calibrator)_. (e) The relative expression of miR‐125a was proceeded via calculating 2^−ΔΔ^ *^C^* ^t^. Primers were listed as follows: lnc‐MALAT1, forward: TCCTAAGGTCAAGAGAAGTGTCAG, reverse: GTGGCGATGTGGCAGAGAA; miR‐125a, forward: ACACTCCAGCTGGGTCCCTGAGACCCTTTA, reverse: TGTCGTGGAGTCGGCAATTC; GAPDH, forward: GAGTCCACTGGCGTCTTCAC, reverse: ATCTTGAGGCTGTTGTCATACTTCT; and U6, forward: CTCGCTTCGGCAGCACA, reverse: AACGCTTCACGAATTTGCGT.

### 2.7 28‐day mortality calculation

According to the patient's presenting illness and local patterns of infection*,* different treatments such as antimicrobial therapy, antiviral therapy, or combination therapy were given to them after admission. During hospitalization, daily follow‐up was conducted for all sepsis patients until they died in hospital or 28 days after enrollment. The death event was recorded during follow‐up, and all patients were further classified as deaths and survivors. Accumulating mortality was calculated from the date of enrollment to the date of death or censored to the date of last follow‐up.

### 2.8 Statistical analysis

Statistical analysis was performed using SPSS version 24.0 (IBM), and figure was plotted with the use of GraphPad Prism version 7.01 (GraphPad Software). Normally distributed continuous data were displayed as mean ± standard deviation (SD), and non‐normally distributed continuous data were expressed as median and interquartile range (IQR). Categorical data were presented as count and percentage. Student's *t* test was used to assess the statistical significance of normally distributed continuous data between two groups, while Wilcoxon's rank sum test was used to assess the statistical significance of non‐normally distributed continuous data between two groups. Chi‐square test was used to compare the proportions of categorical data between two groups. Spearman's rank correlation test was used to evaluate the correlation between two continuous variables. Receiver operating characteristic (ROC) curve and the area under the curve (AUC) with 95% confidence interval (CI) were used to assess the performance of variables in distinguishing sepsis patients from healthy controls and 28‐day mortality risk. The Kaplan‐Meier curve was plotted to display the accumulating mortality. Log‐rank test was used to analyze the statistical significance of accumulating mortality between two groups. A two‐sided *P* value < .05 was considered statistically significant.

## 3 RESULTS

### 3.1 Clinical characteristics

The age of sepsis patients and healthy controls was 58.2 ± 11.2 years and 57.1 ± 12.1 years, respectively (Table 1). The number of females and males was 66 (33.7%) and 130 (66.3%), respectively, in sepsis patients, and 77 (39.3%) and 119 (60.7%) respectively, in healthy controls. There was no difference in age (*P* = .355) and gender (*P* = .248) between sepsis patients and healthy controls. Scr (*P* < .001), WBC (*P* = .019), and CRP (*P* < .001) were increased, while albumin (*P* < .001) was decreased in sepsis patients compared with healthy controls. Other detailed clinical characteristics of sepsis patients and healthy controls were shown in Table 1.

**Table 1:** Clinical features

Items	Sepsis patients (N = 196)	Healthy controls (N = 196)	*P* value
Age (years)			
Mean ± SD	58.2 ± 11.2	57.1 ± 12.1	.355
Range	32.0‐80.0	28.0‐79.0	
Gender, No. (%)			
Female	66 (33.7)	77 (39.3)	.248
Male	130 (66.3)	119 (60.7)	
BMI (kg/m^2^)			
Mean ± SD	22.5 ± 3.7	22.6 ± 3.2	.795
Range	15.6‐31.0	16.1‐30.1	
Scr (mg/dL)			
Median (IQR)	1.7 (1.2‐2.4)	0.8 (0.7‐1.0)	<.001
Range	0.6‐8.4	0.5‐1.3	
Albumin (g/L)			
Median (IQR)	26.9 (21.4‐36.8)	42.6 (39.5‐45.6)	<.001
Range	15.2‐62.9	29.0‐60.3	
WBC (*10^9^/L)			
Median (IQR)	11.5 (2.5‐26.5)	6.4 (5.3‐7.7)	.019
Range	0.4‐72.0	3.5‐11.6	
CRP (mg/L)			
Median (IQR)	99.6 (51.3‐157.9)	3.7 (2.4‐6.2)	<.001
Range	16.7‐574.8	0.3‐11.9	
APACHE II score			
Mean ± SD	13.6 ± 6.0	‐	‐
Range	5.0‐31.0	‐	‐
SOFA score			
Mean ± SD	6.1 ± 2.8	‐	‐
Range	3.0‐14.0	‐	‐
TNF‐α (pg/mL)			
Median (IQR)	198.0 (114.9‐314.3)	‐	‐
Range	13.2‐981.1	‐	‐
IL‐1β (pg/mL)			
Median (IQR)	8.6 (4.2‐19.5)	‐	‐
Range	1.3‐115.8	‐	‐
IL‐6 (pg/mL)			
Median (IQR)	84.4 (42.1‐177.2)	‐	‐
Range	5.7‐885.2	‐	‐
IL‐8 (pg/mL)			
Median (IQR)	127.3 (51.1‐208.4)	‐	‐
Range	9.4‐1079.3	‐	‐

Abbreviations: APACHE II, acute pathologic and chronic health evaluation II; BMI, body mass index; COPD, chronic obstructive pulmonary disease; CRP, C‐reactive protein; IL, interleukin; IQR, interquartile range; Scr, serum creatinine; SD, standard deviation; SOFA, Sequential Organ Failure Assessment; TNF‐α, tumor necrosis factor‐α; WBC, white blood cell.

### 3.2 Lnc‐MALAT1/miR‐125a axis, lnc‐MALAT1, and miR‐125a in sepsis

**Figure 1:**
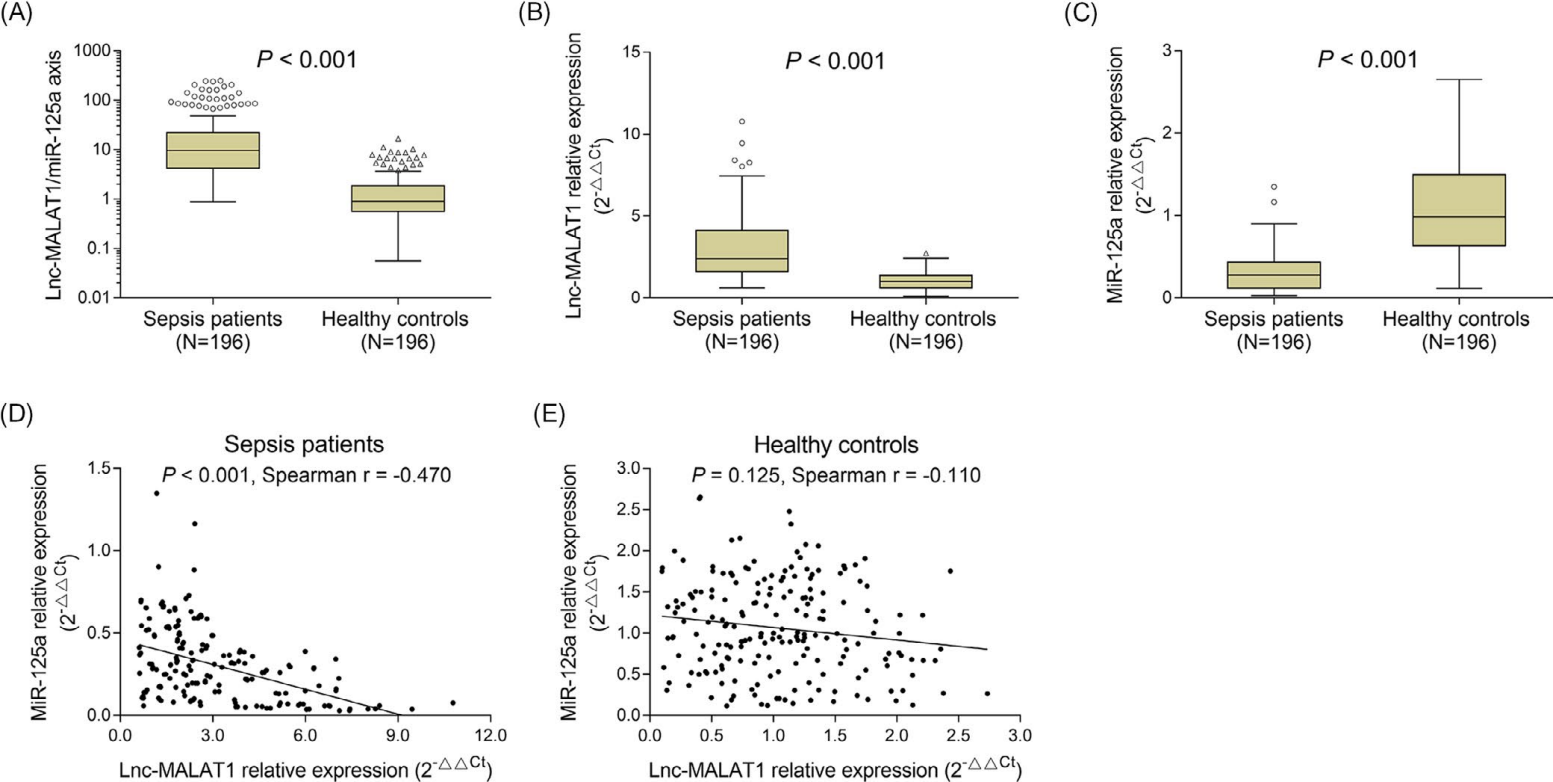
Lnc‐MALAT1/miR‐125a axis, lnc‐MALAT1, and miR‐125a in sepsis patients and healthy controls. Comparison of lnc‐MALAT1/miR‐125a axis (A), lnc‐MALAT1 (B), and miR‐125a (C) between sepsis patients and healthy controls (A). Correlation of miR‐125a with lnc‐MALAT1 in sepsis patients (D) and healthy controls (E). Lnc‐MALAT1, long non‐coding RNA metastasis‐associated lung adenocarcinoma transcript 1; miR‐125a, microRNA 125a

Lnc‐MALAT1/miR‐125a axis was increased in sepsis patients (9.713 [4.217‐22.037]) compared with healthy controls (0.905 [0.566‐1.852]) (*P* < .001) (Figure 1A). Lnc‐MALAT1 relative expression was elevated in sepsis patients (2.397 [1.600‐4.118]) compared with healthy controls (1.011 [0.613‐1.375]) (*P* < .001) (Figure 1B), while miR‐125a relative expression was decreased in sepsis patients (0.276 [0.120‐0.435]) compared with healthy controls (0.986 [0.635‐1.495]) (*P* < .001) (Figure 1C). The correlation of miR‐125a with lnc‐MALAT1 was further analyzed in sepsis patients and healthy controls, respectively, which observed that lnc‐MALAT1 relative expression was negatively associated with miR‐125a relative expression in sepsis patients (*r* = −.470, *P* < .001) (Figure 1D), while there was no association between lnc‐MALAT1 relative expression and miR‐125a in healthy controls (*r* = −.110, *P* = .125) (Figure 1E).

### 3.3 Performance of lnc‐MALAT1/miR‐125a axis, lnc‐MALAT1, and miR‐125a in discriminating sepsis patients from healthy controls

**Figure 2:**
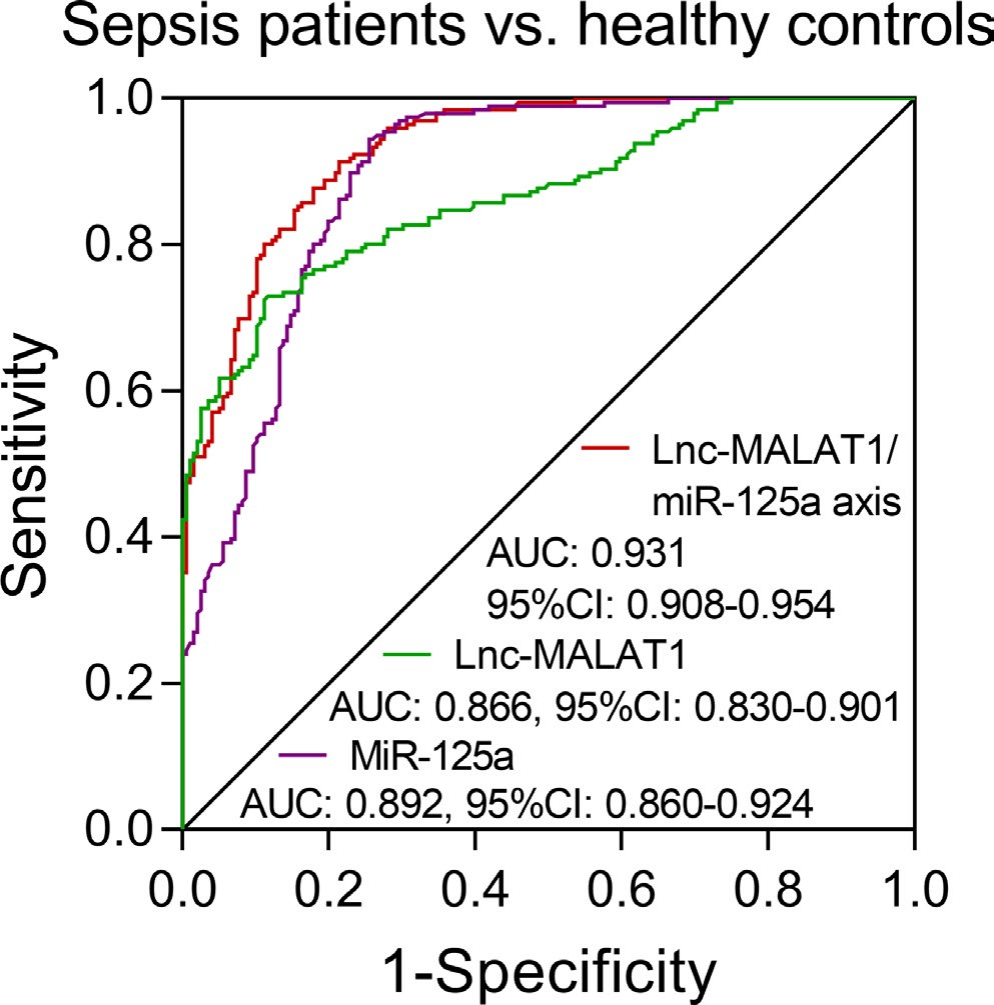
The discriminative value of lnc‐MALAT1/miR‐125a axis, lnc‐MALAT1, and miR‐125a. Lnc‐MALAT1, long non‐coding RNA metastasis‐associated lung adenocarcinoma transcript 1; miR‐125a, microRNA 125a

To further detect the performance of lnc‐MALAT1/miR‐125a axis, lnc‐MALAT1, and miR‐125a in discriminating sepsis patients from healthy controls, we performed ROC curve and observed that lnc‐MALAT1/miR‐125a axis was of excellent value in distinguishing sepsis patients from healthy controls with AUC of 0.931 (95% CI: 0.908‐0.954) (Figure 2). The sensitivity, specificity, negative predictive value (NPV), and positive predictive value (PPV) at best cutoff point (the point where the largest sum of sensitivity and specificity occurred) were 91.3%, 78.6%, 90.1%, and 81.0%, respectively (Table S1). Meanwhile lnc‐MALAT1 (AUC: 0.866, 95% CI: 0.830‐0.901) and miR‐125a (AUC: 0.892, 95% CI: 0.860‐0.924) exhibited good value in discriminating sepsis patients from healthy controls as well. The sensitivity, specificity, NPV, and PPV at best cutoff point of lnc‐MALAT1 and miR‐125a were shown in Table S1. Furthermore, lnc‐MALAT1/miR‐125a axis presented numerically higher value of AUC compared with lnc‐MALAT1 and miR‐125a, suggesting the increased value of lnc‐MALAT1/miR‐125a axis in distinguishing sepsis patients from healthy controls.

### 3.4 Correlation of lnc‐MALAT1/miR‐125a axis, lnc‐MALAT1, and miR‐125a with APACHE II score and SOFA score in sepsis patients

**Figure 3:**
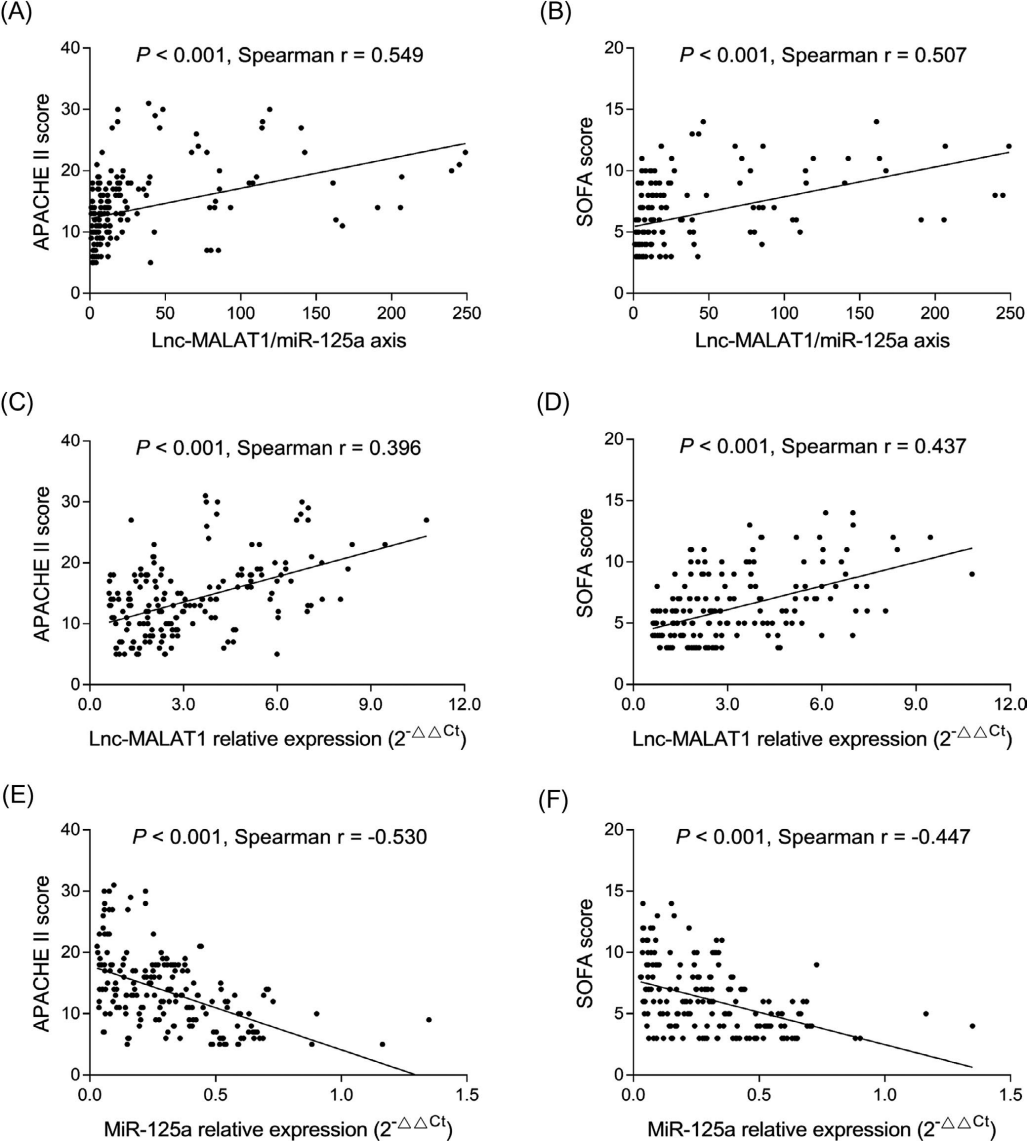
Correlation of lnc‐MALAT1/miR‐125a axis, lnc‐MALAT1, and miR‐125a with general disease severity in sepsis patients. Correlation of lnc‐MALAT1/miR‐125a axis with APACHE II score (A) and SOFA (B) score in sepsis patients. Correlation of lnc‐MALAT1 with APACHE II score (C) and SOFA (D) score in sepsis patients. Correlation of miR‐125a axis with APACHE II score (E) and SOFA (F) score in sepsis patients. Lnc‐MALAT1, long non‐coding RNA metastasis‐associated lung adenocarcinoma transcript 1; miR‐125a, microRNA 125a; APACHE II, acute pathologic and chronic health evaluation II; and SOFA, Sequential Organ Failure Assessment

In sepsis patients, lnc‐MALAT1/miR‐125a axis was positively associated with APACHE II score (*r* = .549, *P* < .001) (Figure 3A) and SOFA score (*r* = .507, *P* < .001) (Figure 3B). Lnc‐MALAT1 relative expression was positively correlated with APACHE II score (*r* = .396, *P* < .001) (Figure 3C) and SOFA score (*r* = .437, *P* < .001) (Figure 3D). However, miR‐125a relative expression was negatively correlated with APACHE II score (*r* = −.530, *P* < .001) (Figure 3E) and SOFA score (*r* = −.447, *P* < .001) (Figure 3F).

### 3.5 Correlation of lnc‐MALAT1/miR‐125a axis, lnc‐MALAT1, and miR‐125a with biochemical indexes and inflammatory cytokines in sepsis patients

Lnc‐MALAT1/miR‐125a axis was positively associated with Scr (*r* = .259, *P* < .001), CRP (*r* = .507, *P* < .001), TNF‐α (*r* = .413, *P* < .001), IL‐1β (*r* = .409, *P* < .001), IL‐6 (*r* = .409, *P* < .001), and IL‐8 (*r* = .421, *P* < .001), while negatively associated with albumin (*r* = −.307, *P* < .001) in sepsis patients (Table 2). Lnc‐MALAT1 was positively correlated with Scr (*r* = .254, *P* < .001), CRP (*r* = .494, *P* < .001), TNF‐α (*r* = .387, *P* < .001), IL‐1β (*r* = .330, *P* < .001), IL‐6 (*r* = .431, *P* < .001), and IL‐8 (*r* = .420, *P* < .001), while negatively associated with albumin (*r* = −.153, *P* = .033) in sepsis patients. MiR‐125a was negatively associated with Scr (*r* = −.261, *P* < .001), WBC (*r* = −.184, *P* = .010), CRP (*r* = −.412, *P* < .001), TNF‐α (*r* = −.387, *P* < .001), IL‐1β (*r* = −.402, *P* < .001), IL‐6 (*r* = −.309, *P* < .001), and IL‐8 (*r* = −.316, *P* < .001), while positively correlated with albumin (*r* = .398, *P* < .001) in sepsis patients.

**Table 2:** Correlation of lnc‐MALAT1/miR‐125a axis, lnc‐MALAT1, and miR‐125a with common biochemical indexes and inflammatory cytokines in sepsis patients

Items	Lnc‐MALAT1/miR‐125a axis		Lnc‐MALAT1		MiR‐125a	
	*P* value	Spearman r	*P* value	Spearman r	*P* value	Spearman r
Scr	<.001	0.259	<.001	0.254	<.001	−0.261
Albumin	<.001	−0.307	.033	−0.153	<.001	0.398
WBC	.046	0.143	.132	0.108	.010	−0.184
CRP	<.001	0.507	<.001	0.494	<.001	−0.412
TNF‐α	<.001	0.413	<.001	0.387	<.001	−0.347
IL‐1β	<.001	0.412	<.001	0.330	<.001	−0.402
IL‐6	<.001	0.409	<.001	0.431	<.001	−0.309
IL‐8	<.001	0.421	<.001	0.420	<.001	−0.316

Abbreviations: CRP, C‐reactive protein; IL, interleukin; Lnc‐MALAT1, long non‐coding RNA MALAT1; miR‐125a, microRNA‐125a; Scr, serum creatinine; TNF‐α, tumor necrosis factor‐α; WBC, white blood cell.

### 3.6 Correlation of lnc‐MALAT1/miR‐125a axis, lnc‐MALAT1, and miR‐125a with mortality in sepsis patients

**Figure 4:**
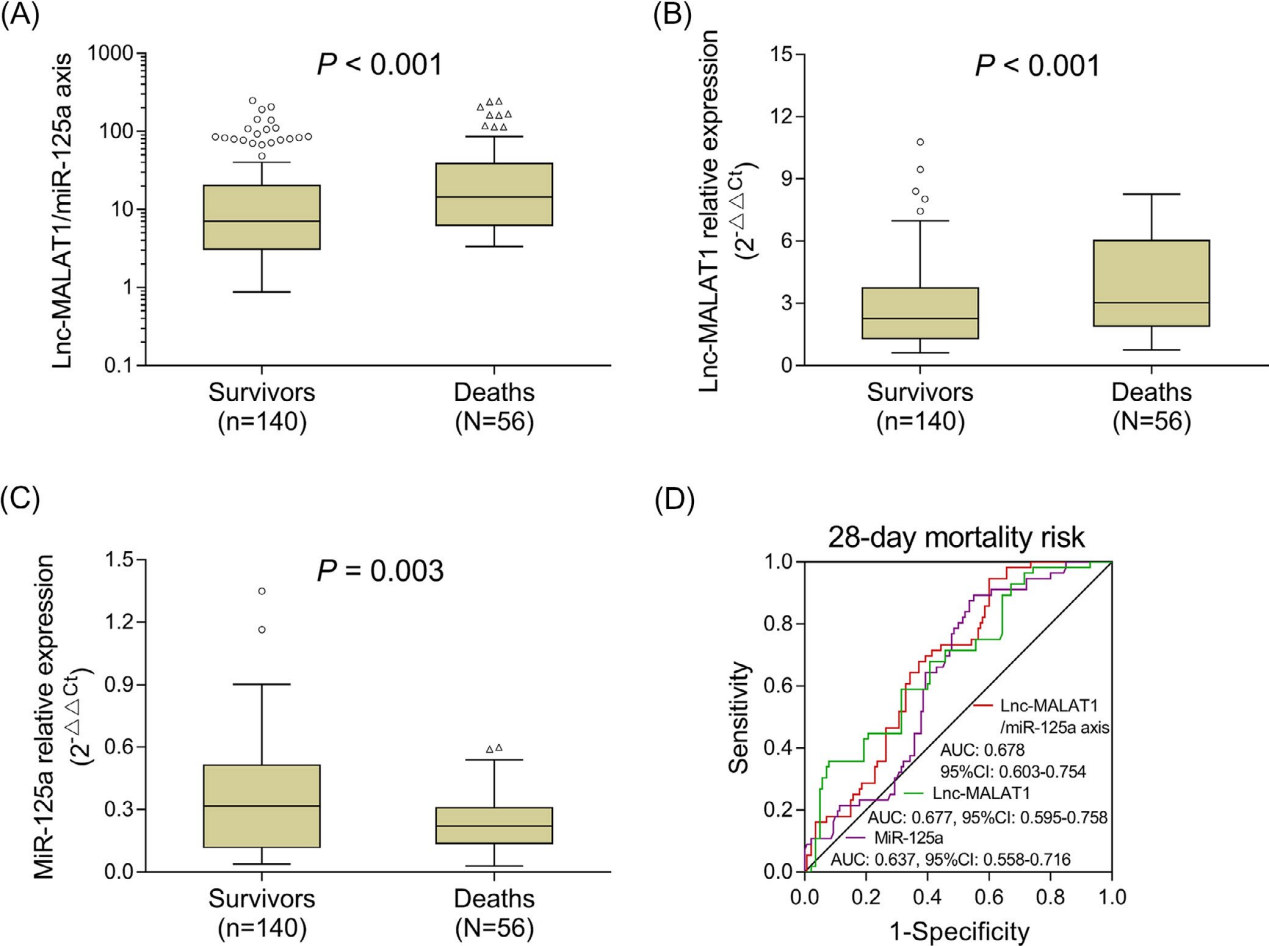
The value of lnc‐MALAT1/miR‐125a axis, lnc‐MALAT1, and miR‐125a in predicting 28‐d mortality risk in sepsis patients. Comparison of lnc‐MALAT1/miR‐125a axis (A), lnc‐MALAT1 relative expression (B), and miR‐125a (C) between survivors and deaths in sepsis patients. Value of lnc‐MALAT1/miR‐125a axis, lnc‐MALAT1 relative expression, and miR‐125a in predicting 28‐day mortality risk (D). Lnc‐MALAT1, long non‐coding RNA metastasis‐associated lung adenocarcinoma transcript 1; miR‐125a, microRNA 125a; AUC, area under the curve; and CI: confidence interval

During hospitalization, daily follow‐up was performed for all sepsis patients until they died in hospital or 28 days after enrollment, and all patients were further classified as deaths (n = 56) and survivors (n = 140). In sepsis patients, lnc‐MALAT1/miR‐125a axis was decreased in survivors (7.048 [3.112‐20.334]) compared with deaths (14.408 [6.287‐38.709]) (*P* < .001) (Figure 4A), and lnc‐MALAT1 relative expression was also reduced in survivors (2.266 [1.311‐3.738]) compared with deaths (3.026 [1.901‐6.032]) (*P* < .001) (Figure 4B). However, miR‐125a relative expression was increased in survivors (0.317 [0.118‐0.513]) compared with deaths (0.221 [0.138‐0.308]) (*P* = .003) (Figure 4C). In addition, ROC curve was conducted to assess the performance of lnc‐MALAT1/miR‐125a axis, lnc‐MALAT1, and miR‐125a in predicting 28‐day mortality risk. We found that lnc‐MALAT1/miR‐125a axis (AUC:0.678, 95% CI: 0.603‐0.754), lnc‐MALAT1 (AUC: 0.677, 95% CI: 0.595, 0.758), and miR‐125a (AUC: 0.637, 95% CI: 0.558, 0.716) could predict 28‐day mortality risk to some extent (Figure 4D) Furthermore, the more detailed information (including sensitivity, specificity, NPV, and PPV) of best cutoff point were shown in Table S1.

### 3.7 Correlation of lnc‐MALAT1/miR‐125a axis, lnc‐MALAT1, and miR‐125a with accumulating mortality in sepsis patients

**Figure 5:**
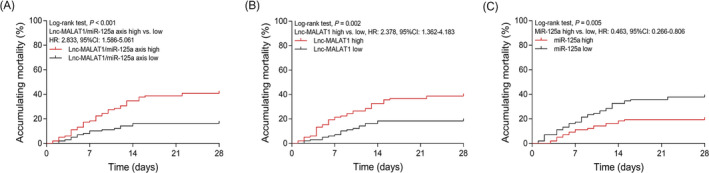
Correlation of lnc‐MALAT1/miR‐125a axis, lnc‐MALAT1, and miR‐125a with mortality in sepsis patients. Comparison of accumulating mortality between sepsis patients with high lnc‐MALAT1/miR‐125a axis and sepsis patients with low lnc‐MALAT1/miR‐125a axis (A). Comparison of accumulating mortality between sepsis patients with high lnc‐MALAT1 expression and sepsis patients with low lnc‐MALAT1 expression (B). Comparison of accumulating mortality between sepsis patients with high miR‐125a expression and sepsis patients with low miR‐125a expression (C). Lnc‐MALAT1, long non‐coding RNA metastasis‐associated lung adenocarcinoma transcript 1; miR‐125a, microRNA 125a

According to the median level of lnc‐MALAT1/miR‐125a axis, lnc‐MALAT1, or miR‐125a, all sepsis patients were divided into those with high level and low level, respectively. Accumulating mortality was increased in patients with lnc‐MALAT1/miR‐125a axis high compared with those with lnc‐MALAT1/miR‐125a axis low (HR = 2.833, 95% CI: 1.586‐5.061, *P* < .001) (Figure 5A). Accumulating mortality was also elevated in patients with lnc‐MALAT1 high expression compared with those with low expression (HR = 2.378, 95% CI: 1.362‐4.183, *P* = .002) (Figure 5B), while was decreased in patients with miR‐125a high expression compared with those with low expression (HR = 0.463, 95% CI: 0.266‐0.806) (Figure 5C).

## 4 DISCUSSION

In the present study, we found that (a) lnc‐MALAT1/miR‐125a axis was upregulated in sepsis patients compared with healthy controls and presented with excellent value in distinguishing sepsis patients from healthy controls. (b) In sepsis patients, lnc‐MALAT1/miR‐125a axis was positively associated with general disease severity, organ injury, and inflammation level. (c) High lnc‐MALAT1/miR‐125a axis predicted increased 28‐day mortality in sepsis patients. (d) Lnc‐MALAT1/miR‐125a axis presented superior value in distinguishing sepsis patients from healthy controls, closer association with general disease severity, organ injury, and inflammation level, but similar performance in predicting 28‐day mortality risk compared with lnc‐MALAT1 or miR‐125a alone.

Lnc‐MALAT1 is indicated to participate in kinds of physiological processes, and recent several papers exhibit that lnc‐MALAT1 promotes inflammatory responses via its interaction with some inflammation‐related signaling pathways, which leads to LPS‐induced injury in septic disease.[6, 7, 8, 9, 19, 20] For example, the research of septic cardiomyocytes demonstrates that lnc‐MALAT1 overexpression presents enhancing effect on TNF‐α expression via activating serum amyloid antigen 3, which aggravates LPS‐induced cell apoptosis and further leads to heavier damage to myocardial tissue.[6] In another study, lnc‐MALAT1 acts as a pro‐inflammatory factor through increasing the expression of myeloid differentiation factor 88, TNF‐α, IL‐1β, and IL‐6 and inhibiting nuclear factor‐κB (NF‐κB) signaling pathway, promoting inflammatory response in LPS‐induced septic acute lung injury.[8] In addition, lnc‐MALAT1 is identified to present with complementary sequence of miR‐125a.[10] Regarding miR‐125a, it is shown to play an important role in the regulation of inflammatory response and possess anti‐inflammation effect against inflammation‐induced injuries.[12, 15] For example, miR‐125a is positively associated with immunosuppression via activating M2 polarization in macrophages, further contributing to cell‐protective effects and tissue repair in inflammation response.[12] As for the role of miR‐125a in sepsis, one previous study indicates that miR‐125a expression was decreased in sepsis patients compared with healthy controls and negatively associated with disease severity scale score, pro‐inflammatory cytokine level, and decreased mortality in sepsis patients.[15] Based on the previous studies, we hypothesized that lnc‐MALAT1/miR‐125a axis might be of value in discriminating sepsis patients from healthy controls and correlated with disease severity, inflammation level, and survival profile in sepsis patients. However, the role of lnc‐MALAT1/miR‐125a axis has not been studies in sepsis yet. In the present study, we found that lnc‐MALAT1/miR‐125a axis was upregulated in sepsis patients compared with healthy controls and presented with excellent value in distinguishing sepsis patients from healthy controls. And in sepsis patients, lnc‐MALAT1 relative expression was negatively associated with miR‐125a relative expression. The possible explanation behind might include that according to the previous studies, lnc‐MALAT1 might enhance phagocytic and bactericidal activities via increasing production of inflammation factors and activating inflammatory responses, while miR‐125a might have opposite effect via promoting macrophage M2 functionality.[8, 12] Furthermore, miR‐125a functions as sponge of lnc‐MALAT1.[10] Therefore, upregulation of lnc‐MALAT1/miR‐125a axis might cause the higher level of inflammation, inducing LPS‐evoked cell damage and tissue injury; therefore, lnc‐MALAT1/miR‐125a axis exhibited increased value in distinguishing sepsis patients from healthy controls.

Following that, we further detected the association of lnc‐MALAT1/miR‐125a axis with APACHE II score, SOFA score, biochemical indexes, and inflammatory cytokines in sepsis patients. We found that lnc‐MALAT1/miR‐125a axis was positively associated with APACHE II score, SOFA score, Scr, CRP, TNF‐α, IL‐1β, IL‐6, and IL‐8, while negatively associated with albumin in sepsis patients. This observation was consistent with the results of existing evidence that lnc‐MALAT1 was positively correlated with Scr, WBC, CRP, TNF‐α, IL‐8, IL‐17, APACHE II score, and SOFA score, while miR‐125 showed the opposite trend with these inflammatory cytokines and disease severity in sepsis patients.[15, 17] The possible reason might be that (a) according to one previous study, lnc‐MALAT1 might act as a regulator of NF‐κB signaling pathway, and inhibition of miR‐125a might stimulate classical activation of macrophages but suppress alternative activation polarization of macrophages. Therefore, increased expression of lnc‐MALATA1/miR‐125a axis might elevate the pro‐inflammatory cytokines level, but decrease the anti‐inflammatory cytokines level.[20, 21] (b) In addition, lnc‐MALAT1/miR‐125a axis high expression might increase the production of pro‐inflammatory cytokines via mediating pro‐inflammatory transcriptional signaling, further leading to stimulation of LPS‐induced cell apoptosis and exacerbated septic organ injury, which contributed elevated general disease severity and organ injuries in sepsis patients.

In addition, we analyzed the association of lnc‐MALAT1/miR‐125a axis with survival profiles in sepsis patients and observed that lnc‐MALAT1/miR‐125a axis was of value in predicting 28‐day mortality risk. The possible reason might be that based on our previous finding that lnc‐MALAT1/miR‐125a axis was associated with increased general disease severity, organ injury, and inflammation level in sepsis patients; hence, sepsis patients with higher lnc‐MALAT1/miR‐125a axis were more vulnerable to multiple organ dysfunction and poor prognosis in sepsis. Interesting, we also found that lnc‐MALAT1/miR‐125a axis presented superior value in distinguishing sepsis patients from healthy controls, closer association with general disease severity, organ injury, and inflammation level, but similar performance in predicting 28‐day mortality risk compared with lnc‐MALAT1 or miR‐125a alone.

There were some limitations in our study. (a) We detected the value of lnc‐MALAT1/miR‐125a axis in predicting 28‐day mortality risk, while the correlation of lnc‐MALAT1/miR‐125a with long‐term clinical outcomes in sepsis patients needed further exploration. (b) Our present study did not include the underlying mechanism of lnc‐MALAT1/miR‐125a axis in LPS‐induced cell apoptosis and septic tissue damage; therefore, further functional experiments were needed. (c) The present study was a single‐center study with a relative small sample size, which might lead to decreased statistical power; therefore, more patients from multiple regions were needed for validation.

In conclusion, lnc‐MALAT1/miR‐125a axis presents excellent value in differentiating sepsis patients from healthy controls and also exhibits positive association with general disease severity, organ injury, inflammation level, and mortality in sepsis patients, which provides the evidence that lnc‐MALAT1/miR‐125a is of potential value as a combination biomarker in sepsis management.

## CONFLICT OF INTEREST

The authors declare that they have no conflict of interest.
