# Supplementary material for: Long non‐coding RNA MALAT1/microRNA 125a axis presents excellent value in discriminating sepsis patients and exhibits positive association with general disease severity, organ injury, inflammation level, and mortality in sepsis patients
Source: J Clin Lab Anal. 2020 Apr 20;34(6):e23222. doi: 10.1002/jcla.23222 (PMC7307338; doi:10.1002/jcla.23222)
Supplement: Supplementary file 1 [file JCLA-34-e23222-s001.docx]

**Supplementary table 1.** Sensitivity, specificity, NPV and PPV at best cut-off point in ROC curves

| Items | Sensitivity | Specificity | NPV | PPV |
| --- | --- | --- | --- | --- |
| **Discriminative ability** |  |  |  |  |
| Lnc-MALAT1/miR-125a axis | 91.3% | 78.6% | 90.1% | 81.0% |
| Lnc-MALAT1 | 73.0% | 88.3% | 76.5% | 86.1% |
| MiR-125a | 94.4% | 74.5% | 93.0% | 78.7% |
| **28-day mortality risk** |  |  |  |  |
| Lnc-MALAT1/miR-125a axis | 94.6% | 40.0% | 94.9% | 38.7% |
| Lnc-MALAT1 | 35.7% | 92.1% | 78.2% | 64.5% |
| MiR-125a | 89.3% | 45.0% | 91.3% | 39.4% |

NPV, negative predictive value; PPV, positive predictive value; ROC, receiver operating characteristic.
